# EWAS in a polyphenol dense, DNA methylation-targeted, controlled diet and lifestyle study

**DOI:** 10.3389/fnut.2026.1879595

**Published:** 2026-08-20

**Authors:** Kara N. Fitzgerald, Romilly Hodges, Tish Campbell, Natália Carreras-Gallo, Ryan Smith, Vanessa Vendramini, Douglas Hanes, David Cheishvili, Moshe Szyf, Ryan Bradley, Varun B. Dwaraka

**Affiliations:** 1 Institute for Functional Medicine, Federal Way, WA, United States; 2 College of Nutrition, Sonoran University of Health Sciences, Tempe, AZ, United States; 3 Helfgott Research Institute, National University of Natural Medicine, Portland, OR, United States; 4 Virginia Commonwealth University, Richmond, VA, United States; 5 TruDiagnostic Inc., Lexington, KY, United States; 6 Laboratory of Reproductive and Developmental Biology (LABReD), Department of Morphology and Genetics, Paulista School of Medicine, Federal University of Sao Paolo – EPM/UNIFESP, Sao Paulo, Brazil; 7 Herbert Wertheim School of Public Health and Human Longevity Sciences, University of California, San Diego, San Diego, CA, United States; 8 Gerald Bronfman Department of Oncology, McGill University, Montreal, QC, Canada; 9 EpiMedTech Global Ltd., Singapore, Singapore; 10 Zhejiang University-Lishui Joint Innovation Center for Life and Health & Lishui Lvgu Institute for Life and Health, Lishui, China

**Keywords:** aging, DNA methylation, epigenome-wide association study, nutrition, polyphenols

## Abstract

**Background:**

Dietary and lifestyle factors can influence DNA methylation patterns. We previously reported epigenetic age attenuation following a controlled study using an 8-week polyphenol-dense, DNA methylation-targeted diet and lifestyle intervention in healthy males (Methylation Diet and Lifestyle Study), with phytonutrient/polyphenol-rich foods (green tea, oolong tea, curcumin, garlic, and berries) being most predictive of this effect.

**Methods:**

Here we conducted an epigenome-wide association study (EWAS) in 38 participants from the Methylation Diet and Lifestyle Study. The intervention included a dietary pattern intentionally rich in substrate and cofactor nutrients for methylation pathways, and components known to alter DNA-methyltransferase (DNMT) enzyme activity. In line with prior EWAS studies with small sample sizes where FDR-significant findings are unlikely, we used pre-specified nominal *P*-value thresholds (0.001, 0.0001) for the exploratory analyses.

**Results:**

At *P* < 0.001 (unadjusted), 676 differentially methylated loci (DML) were identified in the intervention group versus 286 in controls. At *P* < 0.0001 (unadjusted), 50 DML were identified in the intervention group compared to 13 in controls. Fifteen DML were in transcription start site-proximal regions of genes including those involved in zinc homeostasis and nutrient sensing, development and pluripotency, proteostasis and genome stability, tumor suppression, and synaptic function. A group-by-time interaction analysis identified 70 intervention-specific DML at *P* < 0.0001, with nominal enrichment including autophagy, mTOR signaling, and chromatin remodeling pathways. A regional DMR analysis identified 128 within-group and 129 interaction-specific DMRs. DMR functional enrichment analyses revealed convergent nominal associations with lipid metabolism (alpha-linolenic acid, lipoic acid, biosynthesis of unsaturated fatty acids, PPAR signaling, cholesterol homeostasis), central energy metabolism (TCA cycle, glycolysis/gluconeogenesis, pentose phosphate, pyruvate), and nutrient sensing (PI3K-Akt, mTOR, AMPK, autophagy as well as other pathways). As expected for the limited cohort size and short intervention duration, none of the single CpG findings or enrichment analyses survived multiple test correction and are therefore considered exploratory and hypothesis-generating only.

**Conclusion:**

This EWAS identified a larger number of nominally changing CpGs in the intervention group compared to controls as well as biologically coherent methylation changes. These findings provide mechanistic hypotheses for previously observed epigenetic age attenuation. Replication in larger cohorts, longer intervention durations, and functional validation remain essential.

## Introduction

Multiple EWAS studies indicate that diet and lifestyle factors modify DNA methylation and subsequent gene expression (1). This is strengthening scientific understanding of epigenetic alterations as an important mechanism linking environmental factors with aging and disease risk. A portion of these epigenetic changes may also be captured in various DNA methylation-based “epigenetic clocks,” which attempt to predict age acceleration-related morbidity and mortality based on epigenetic patterns (2). Such epigenetic patterns may reflect both intrinsic or “preprogrammed” aging as well as extrinsic or “exposomic” aging (2, 3). Specific epigenetic modifications can also be inherited and lead to phenotypic alterations in offspring across multiple generations (2).

In addition to epigenetic clock predictors, DNA methylation changes at loci outside of those associated with epigenetic clocks, with subsequent impact on gene expression and phenotype, are also relevant modulators of health outcomes over time. These explorations are still in their infancy with a lack of convergence in differentially methylated CpG sites after purportedly-similar exposures (1). This may reflect imprecision in the definition of exposures, the complexity of multifactorial exposures such as diet, interindividual differences in genomics or microbiota, and/or heterogeneity of data processing and analyses. Yet the importance of this line of inquiry is understood for its potential to improve healthspan, reduce healthcare costs, and address other societal challenges of an aging population (4).

We previously undertook a pilot randomized, controlled trial to investigate the potential for an 8-week DNA methylation-targeted dietary and lifestyle intervention to alter epigenetic patterns and health-related biomarkers in 43 healthy, middle-aged males aged 50–72 (5). Salivary DNA methylation was profiled on the Illumina EPIC array, and the intervention group showed a mean 3.23-year decrease in DNAmAge compared to controls (*P* = 0.018) calculated using the Horvath 2013 DNAmAge clock. This clock was selected for its suitability in the available saliva samples, and it has been proposed to capture intrinsic, cell-type-independent properties of the aging process. By contrast, measures that incorporate age-related changes in blood cell composition are thought to reflect greater extrinsic aging, while more recently developed clocks trained on phenotypic health outcomes and mortality risk may capture a broader spectrum of biological aging (6, 7). Within group DNAmAge change was −2.04 years (*P* = 0.043) for the intervention (8). Phenotypic changes included a 15% increase in serum 5-methyltetrahydrofolate (*P* = 0.004), an important nutrient used in the formation of the universal methyl donor S-adenosylmethionine, a 25% reduction in serum triglycerides (*P* = 0.009), and a weight loss of 4.61 lbs (SD = 7.91) for the intervention compared to an average gain of 0.90 lbs (SD = 3.892) in the control group (9). Factors prioritized in the multimodal intervention were those that provided substrate or cofactors for methylation activity in the body, as well as those indicated in the literature as having the potential to modulate the activity of DNA methyltransferase (DNMT) and ten-eleven translocation (TET) enzymes, responsible for adding and removing (respectively) methyl groups on DNA cytosine residues.

Specific polyphenolic modulators of DNMT enzymes (green tea, oolong tea, turmeric, rosemary, garlic, berries, categorized in the original study as methyl adaptogens along with specific daily targets) were identified as the portion of the intervention most predictive of epigenetic age attenuation after adjusting for weight changes and baseline epigenetic age acceleration (B = −1.12; 95% CI −2.80, −0.08, *P* = 0.016) (9). Polyphenols are consistently found in the dietary patterns of centenarians and are known to influence each of the twelve hallmarks of aging (10). The intervention diet total polyphenol intake was relatively high, averaging an estimated 2908 mg/d, an amount greater than has been reported as typically consumed in traditional Mediterranean diets (11).

A more detailed explanation of the cohort baseline characteristics, intervention, and analysis has been published previously (5). A subsequent case series in women using a very similar intervention supports these findings and suggests wider generalizability beyond the original male-only cohort; mean DNAmAge decreased an average of −4.60 years from baseline (*P* = 0.039) in this group (12). Along with other studies examining the effects of environmental inputs on DNA methylation, these data indicate the potential for dietary and lifestyle factors, and in particular polyphenols, to influence the epigenome (10).

This present secondary EWAS analysis of the original methylation-supportive diet and lifestyle pilot trial sought to move beyond global epigenetic age estimates and to explore pathways, gene sets, and specific CpG loci that changed over the 8-week intervention. This analysis comprised data from 38 participants with available paired Baseline and Week 8 saliva samples.

## Materials and methods

### Sample preparation and raw data extraction

Saliva samples (Baseline and Week 8) from 38 participants (18 from the treatment group, 20 from the control group) of the original pilot study (ClinicalTrials.gov Identifier: NCT03472820) were processed at Yale University Center for Genome Analysis and analyzed using the Illumina Methylation EPIC Array Cat. No. WG-317-1001 Illumina Inc., San Diego, CA). Sample placement in each chip was randomized. Raw IDAT files were imported into R and processed using the minfi Bioconductor package. Signal intensities were background-corrected and dye-bias normalized using the single-sample normal-exponential out-of-band (ssNoob) method (minfi preprocessNoob). All raw data files and associated metadata are available in the Keck Microarray Database; study data are also available in the Gene Expression Omnibus (GEO) under accession GSE149747.

### Planned contrasts

We modeled three planned contrasts, including two within-group analyses (within-individual changes from Baseline to Week 8 in both the treatment and control arms), as well as a group-by-time interaction analysis.

### Multiple test correction and use of nominal *P*-value thresholds

False discovery rates (FDR) were calculated using the Benjamini-Hochberg method (via the missMethyl framework) for all differential methylation and functional enrichment analyses. For differential methylation, FDR < 0.05 was considered statistically significant. For functional enrichment, results at both FDR < 0.05 and FDR < 0.1 were both examined. However, given the modest sample size, short intervention duration (8 weeks), and small effect sizes typically observed for lifestyle-induced DNA methylation changes, this pilot study was not powered to detect FDR-significant associations across the full EPIC array (∼866.000 CpGs). Accordingly, no individual CpG results met the thresholds for FDR significance. Therefore, in line with prior small-cohort dietary EWAS studies (13–15), we pre-specified nominal *P*-value thresholds (*P* < 0.001, *P* < 0.0001) for hypothesis generation, visualization of effect direction, and downstream enrichment analyses of differential methylation.

### Within-group analyses

Differential methylation analysis was performed on within-group results using normalized beta values. We used the limma framework to model within-individual changes from Baseline to Week 8 in both the treatment and control arms. Multivariate linear models incorporated fixed effects including beadchip, epithelial cell (Epi), fibroblast (Fib), and immune cell (IC) fractions estimated using EpiDISH with the centEpiFibIC.m 3-class reference; the saliva-appropriate reference that models the three principal cell populations in non-blood tissues; the first three principal components of technical probes, and Study.ID as an individual fixed effect (categorical factor) to control for subject-specific baseline differences. (16). The treatment arm model produced a λ of 0.86, while the control arm yielded a λ of 1.04, indicating that neither analysis demonstrated problematic genomic inflation or deflation. As an additional check against model overfitting, we examined the contribution of included covariates to λ. The final λ values (reported above) did not indicate overfitting or undue compression of the *P*-value distribution, supporting the adequacy of the selected covariate structure. Of the top nominally significant CpG sites, those located in transcription start site-proximal regions were identified. Functional enrichment analyses for the treatment arm results were performed on the top-ranked DMLs using the missMethyl package (version 1.40.3) in R (version 4.4.0) (17). The missMethyl approach was specifically designed for methylation array data and accounts for the variable number of CpG probes per gene, which can bias traditional gene set enrichment methods. KEGG pathway enrichment was tested using the gometh function with the “KEGG” collection parameter, and MSigDB Hallmark gene set enrichment was tested using the gsameth function with Hallmark gene sets downloaded from the Molecular Signatures Database (MSigDB v7.1). For both analyses, the full set of CpG sites on the Illumina EPIC array (*n* = 865,859) was used as the background. Results were visualized as dot plots using ggplot2 (version 4.0.0).

### Group-by-time interaction analysis

To identify methylation changes specific to the intervention rather than shared temporal effects (e.g., study participation effects, seasonal variation, or other within-cohort drift), we conducted a group-by-time interaction contrast analysis using the limma framework, the same covariate structure described above, and nominal *P*-value thresholds of 0.001 and 0.0001. The group-by-time interaction model yielded λ = 1.14 across all 866,553 tested CpGs, reflecting mild inflation consistent with the added complexity of a two-group interaction term in a small cohort. Functional enrichment analysis of interaction CpGs was similarly performed using missMethyl (gometh for KEGG pathways; gsameth for MSigDB Hallmark gene sets v7.1). For the broader *P* < 0.001 set, the full EPIC array (865,859 CpGs) served as the background universe; for the focused *P* < 0.0001 set, the *P* < 0.001 interaction CpGs were used as the background to enable a more specific inference.

### Differentially methylated region (DMR) analysis

To assess whether co-methylated adjacent CpG sites provide regional evidence surviving FDR correction, we performed DMR analysis using DMRcate (version 3.2.1) on the genome-wide limma output from the within-group treatment-arm comparison (Baseline vs. Week 8, *n* = 18 pairs; model: ∼ Test + Age + Epi + Fib + IC, duplicateCorrelation block = SubjectID) and from the group-by-time interaction model described above. CpGs with nominal *P* < 0.001 were used as candidate sites for regional smoothing (kernel bandwidth lambda = 1000 bp, C = 2), with FDR correction applied at the regional level via the Harmonic Mean FDR (HMFDR).

### Cell composition sensitivity analysis

To assess robustness of the within-group findings to longitudinal shifts in salivary cell composition, we performed a sensitivity analysis excluding the five participants (of 18) with the largest total cell-proportion shift from baseline to Week 8 (top quartile by summed absolute change across Epi/Fib/IC fractions; *n* = 13 retained), refitting the same model on the retained subset.

### Cross-study comparison

To assess cross-study reproducibility of intervention-associated methylation changes, the interaction CpGs (*P* < 0.001) were compared with CpGs reported in two independent dietary EWAS: Perlmutter et al. (18) (Tartary buckwheat polyphenol supplement, 90 days) and Dwaraka et al. (13) (vegan vs. omnivore diet, 8 weeks). Overlap was assessed by CpG probe ID matching, with direction of change recorded for overlapping sites. Three-way overlap was visualized using Venn diagrams generated with the VennDiagram R package (v1.7.3).

## Results

### Within-group differential methylation analysis

**FIGURE 1:**
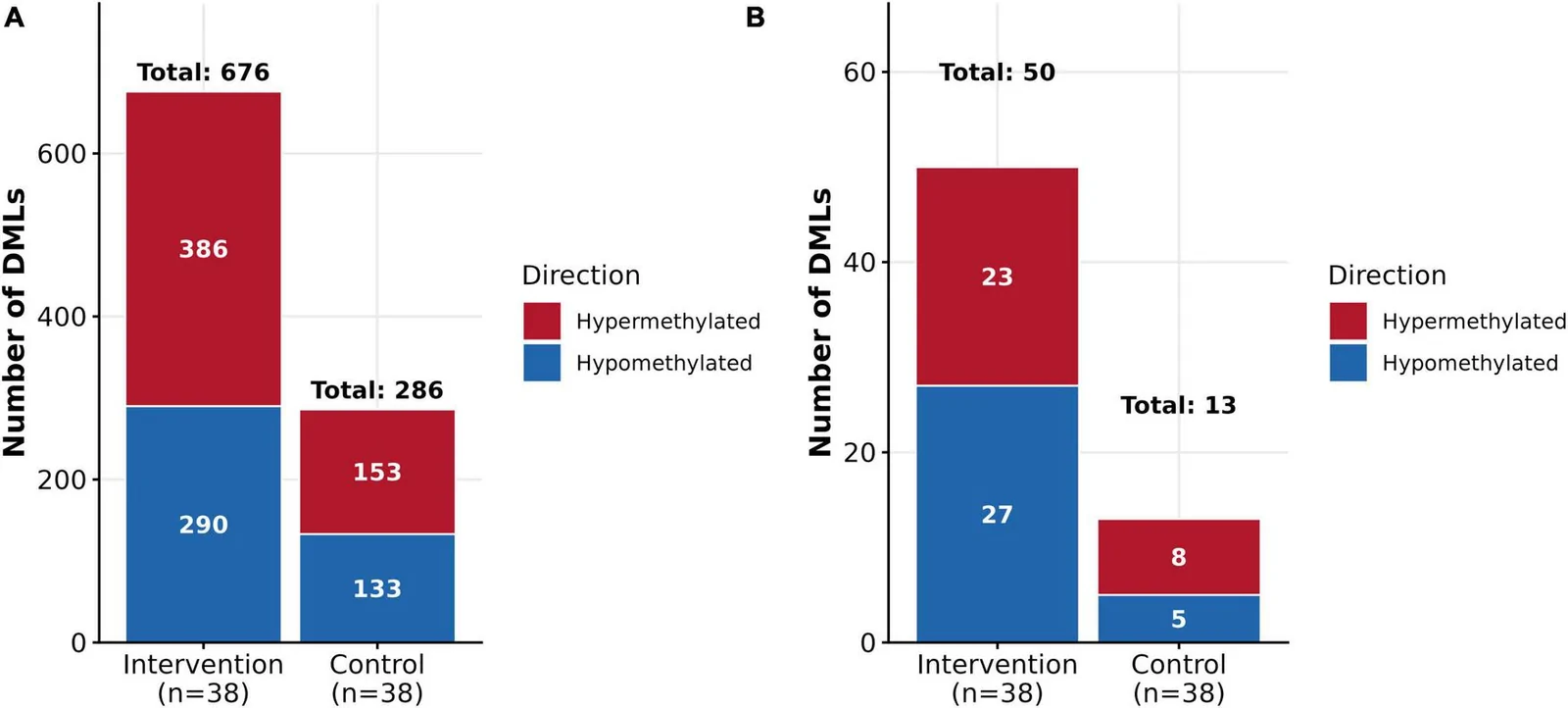
Within-individual differentially methylated loci between baseline and week 8. **(A)** *P* < 0.001 (unadjusted), FDR > 0.05. **(B)** *P* < 0.0001 (unadjusted), FDR > 0.05.

**FIGURE 2:**
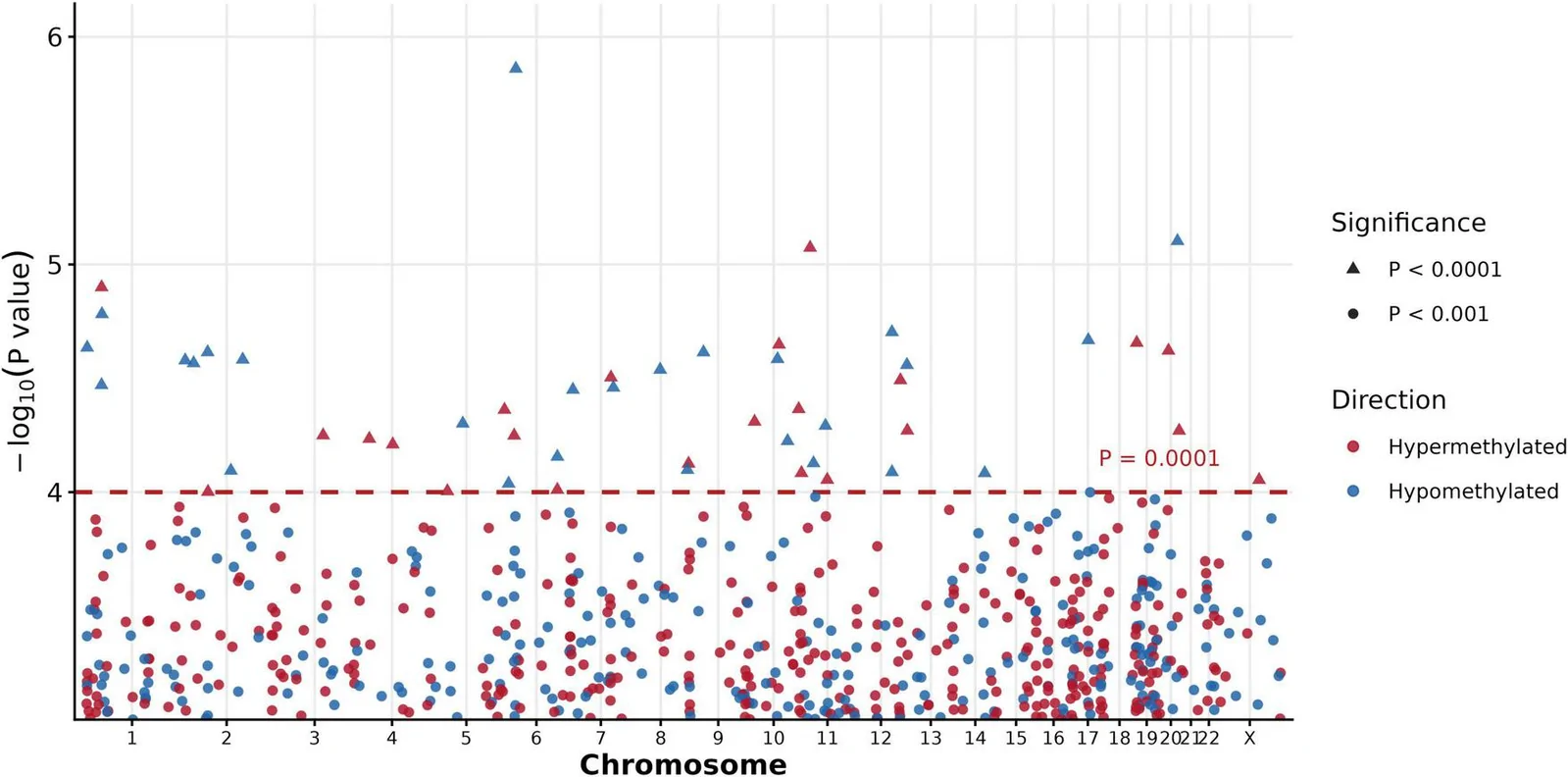
Chromosomal distribution of differentially methylated loci (*P* < 0.0001, *P* < 0.001) in the intervention group. Manhattan plot showing the genomic distribution of DMLs identified in the intervention group (within-individual change, baseline to Week 8). Each point represents a CpG site; its vertical position corresponds to the negative log_10_-transformed unadjusted *P*-value. Chromosomal position is shown on the *x*-axis. Point shape indicates the significance threshold: triangles denote CpGs reaching *P* < 0.0001 and circles denote CpGs at *P* < 0.001. Point color indicates direction of methylation change: red = hypermethylated, blue = hypomethylated. The dashed horizontal line marks the *P* = 0.0001 threshold. DMLs are distributed across all autosomes and the X chromosome, with no single chromosomal region showing dominant enrichment. No CpGs survived FDR correction (FDR > 0.05) in this analysis.

**FIGURE 3:**
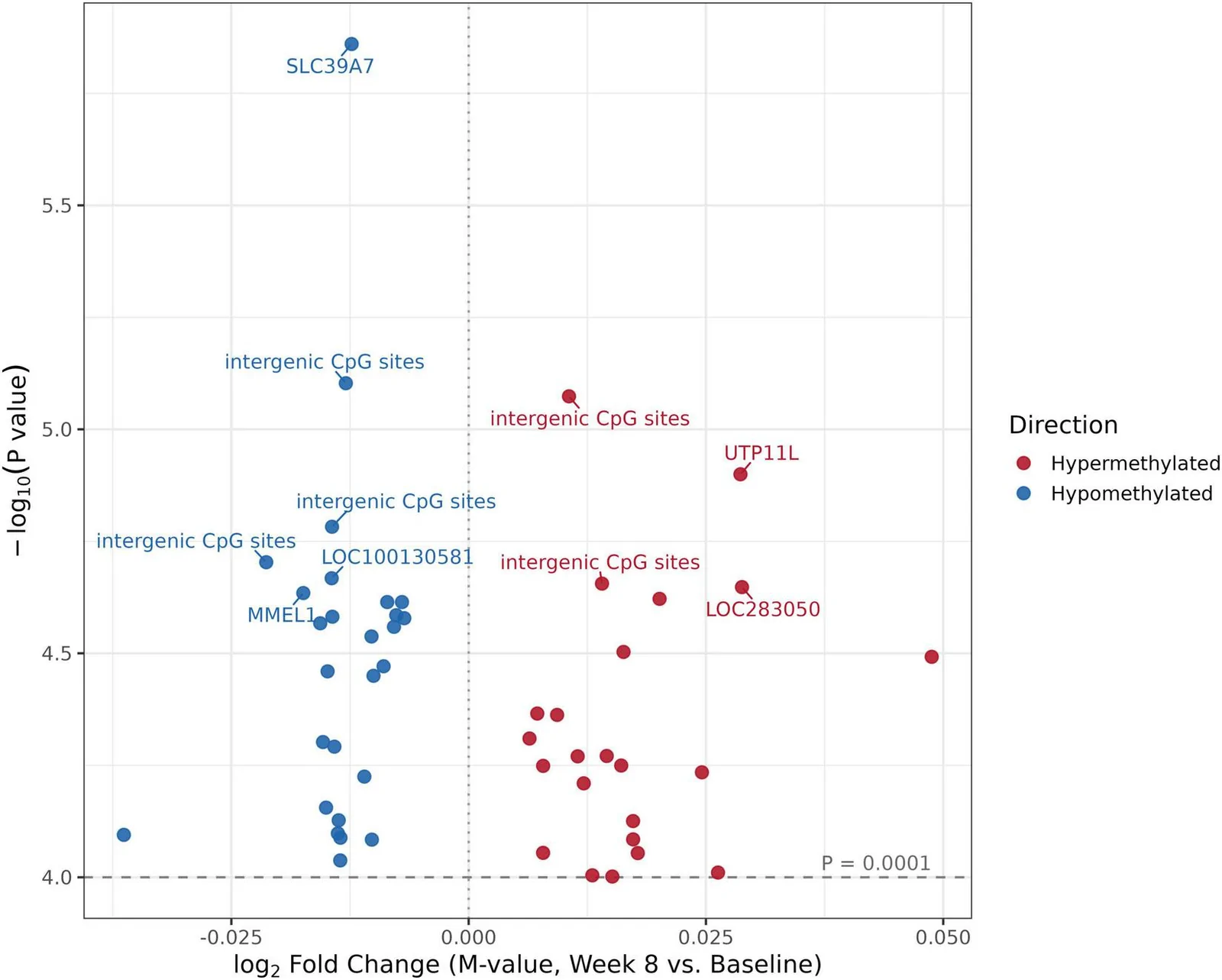
Effect size versus significance for the top differentially methylated loci in the intervention group. This plot displays a filtered subset of the 50 most significantly differentially methylated CpG sites identified in the intervention group (within-individual change, baseline to Week 8; *P* < 0.0001, FDR > 0.05), not the full set of tested CpGs. The x-axis shows the log2 fold change in M-values between Week 8 and baseline, reflecting the magnitude and direction of methylation change. The y-axis shows the negative log10-transformed unadjusted P-value. Red points indicate hypermethylated CpGs (increased methylation at Week 8) and blue points indicate hypomethylated CpGs (decreased methylation at Week 8). The dashed horizontal line marks the *P* = 0.0001 threshold used to select these sites. Selected CpGs are annotated with their nearest gene name, or noted as intergenic where no gene lies nearby. The most significant locus overall maps to SLC39A7, a zinc transporter gene, and is hypomethylated. By effect size, hypermethylated sites show a slightly larger mean and maximum fold change than hypomethylated sites; effect size does not consistently track with significance within either direction.

Using the nominal threshold of *P* < 0.001, 676 within-individual differentially methylated loci (DMLs) were identified in the DNA methylation-targeted diet and lifestyle intervention group, compared to 286 in the control group (Figure 1A). Among the 676 sites, 290 showed hypomethylation and 386 showed hypermethylation. At the nominal threshold of *P* < 0.0001, 50 CpG sites showed within-individual change in the intervention group, compared to 13 in the control group (Figure 1B). Among these 50 sites, 27 exhibited hypomethylation and 23 hypermethylation. The sites identified at the *P* < 0.0001 threshold were additionally visualized using Manhattan and Volcano plots (Figures 2, 3). Genomic inflation factors (λ) were 0.86 for the intervention group and 1.04 for the control group at both *P*-value thresholds, indicating no evidence of test-statistic inflation or deflation. Effect sizes and 95% confidence intervals for the top 676 (*P* < 0.001) and 50 CpG (*P* < 0.0001) sites are provided in Supplementary Tables 1, 2.

#### Exploration of transcription start site-proximal DMLs

Of the 50 DMLs identified in the intervention group at a significance threshold of *P* < 0.0001 and associated with 41 genes, 15 were in transcription start site-proximal regions (TSS200, TSS1500) and may therefore be presumed to have the potential to influence gene expression and phenotype. Twelve sites became less methylated and three more methylated at the Week 8 timepoint. These 15 DMLs were associated with 15 genes (Table 1). Effect sizes with 95% confidence intervals for all 15 CpGs are reported in Table 1; as a representative example, the top-ranked CpG (cg07019243, SLC39A7/RXRB) showed logFC = −0.0123 (95% CI: −0.0167, −0.0079; *P* = 1.38 × 10^−6^), corresponding to a ∼1.2 percentage-point reduction in methylation at Week 8. A forest plot of all 15 TSS-proximal CpGs with 95% CIs is provided as Supplementary Figure 1.

**TABLE 1:** Genes with nominally significant DMLs in transcription start site-proximal regions.

a. Hypo-methylated				
CpG	Gene annotation	Effect (logFC)	95% CI	*P*-value
cg07019243	SLC39A7;RXRB	−0.012328958	(−0.0167, −0.0079)	1.38E-06
cg19367388	UBE2R2	−0.008579551	(−0.0123, −0.0049)	2.43E-05
cg13487762	ZNF503;ZNF503-AS2	−0.007625004	(−0.0109, −0.0043)	2.60E-05
cg23986335	KCNS3	−0.006782945	(−0.0097, −0.0039)	2.64E-05
cg07971474	GEMIN6	−0.015635157	(−0.0224, −0.0089)	2.71E-05
cg05950123	STX2	−0.007868783	(−0.0113, −0.0045)	2.76E-05
cg13987712	MTF1	−0.008961714	(−0.0129, −0.0050)	3.38E-05
cg09189890	BCAP29	−0.014862954	(−0.0214, −0.0083)	3.47E-05
cg21249921	SSBP2	−0.015344591	(−0.0223, −0.0084)	4.99E-05
cg23068057	BCLAF1	−0.015022577	(−0.0220, −0.0081)	6.99E-05
cg15927696	PAX6	−0.013693889	(−0.0201, −0.0073)	7.46E-05
cg08895497	JARID2	−0.013529204	(−0.0199, −0.0071)	9.17E-05
**b. Hyper-methylated**				
**CpG**	**Gene annotation**	**Effect (logFC)**	**95% CI**	***P*-value**
cg14302760	C3orf30;IGSF11	0.016071249	(0.0088, 0.0234)	5.63E-05
cg13777862	KLC2	0.007840854	(0.0041, 0.0115)	8.82E-05
cg00584971	PCDH19	0.017818443	(0.0094, 0.0262)	8.83E-05

(a) Hypomethylated and (b) hypermethylated CpGs (*P* < 0.0001 (unadjusted), FDR > 0.05) located in transcription start site-proximal regions are associated with 15 genes. Effect sizes are shown with 95% confidence intervals (CI). Hypomethylation occurred at 12 CpG sites and hypermethylation occurred at 3 sites.

### Within-group functional enrichment analysis

#### 50 Top-ranked DMLs (*P* < 0.0001, FDR > 0.05) in the intervention group

**FIGURE 4:**
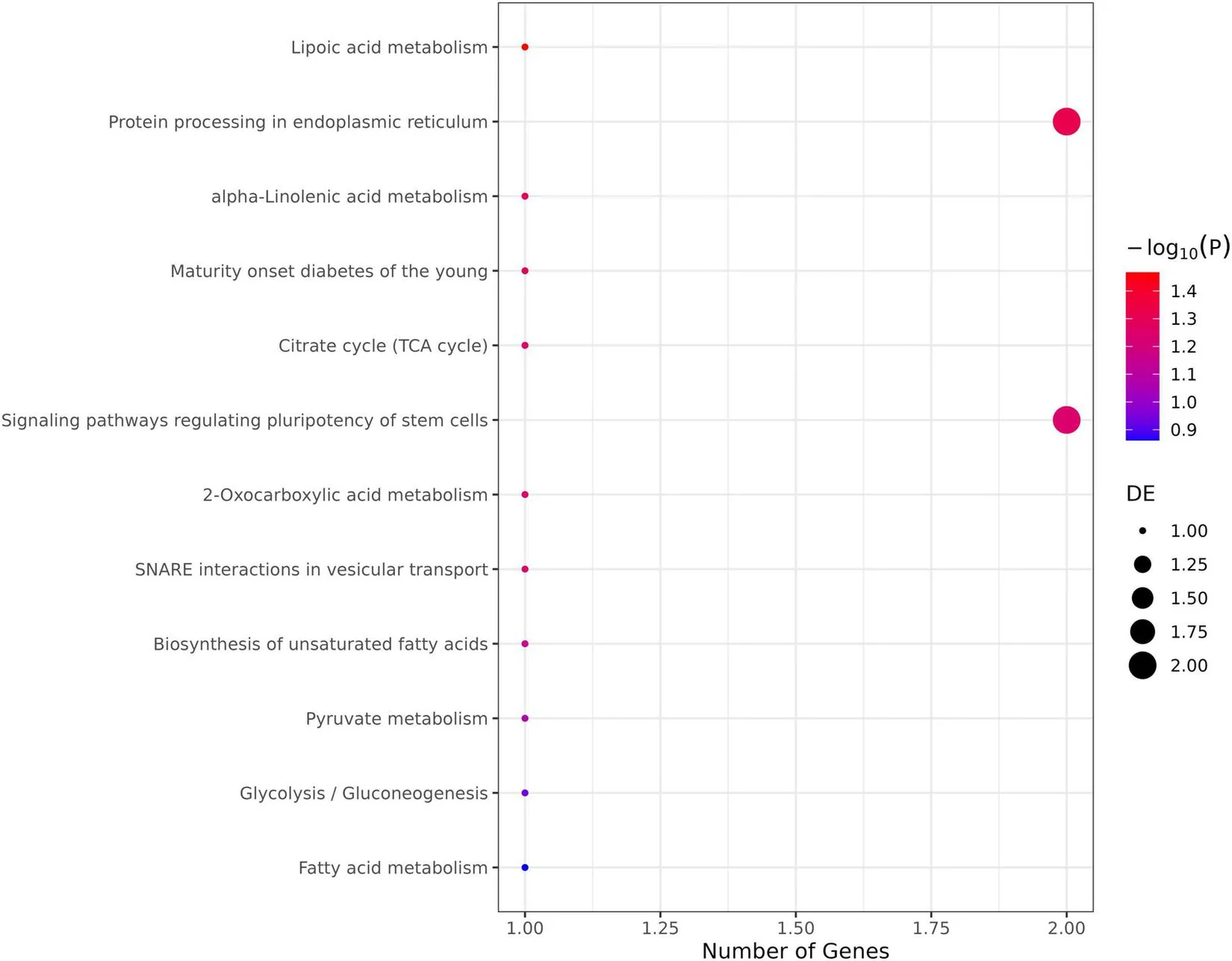
Enriched KEGG pathways for top 50 DMLs (*P* < 0.0001 threshold) in the intervention group (*P* < 0.15 Shown). Dot plot showing the top KEGG pathways enriched among the top 50 significantly differentially methylated CpG sites (within-individual change, baseline to Week 8; *P* < 0.0001, FDR > 0.05). The *x*-axis indicates the number of differentially methylated genes in each pathway, the *y*-axis shows pathway names, dot size represents gene count, and dot color represents the negative log_10_-transformed *P*-value (blue = less significant, red = more significant). Open circles indicate pathways that did not reach FDR < 0.05 after Benjamini-Hochberg correction. Analysis was performed using the missMethyl gometh function to account for probe number bias.

KEGG pathway enrichment analysis of the 50 DMLs identified at the *P* < 0.0001 threshold revealed several metabolic pathways showing nominal enrichment (*P* < 0.1, FDR > 0.05). The most strongly enriched pathway was lipoic acid metabolism (hsa00785; 1 differentially methylated gene; *P* = 0.034), followed by alpha-linolenic acid metabolism (hsa00592; 1 gene; *P* = 0.049) and maturity onset diabetes of the young (hsa04950; 1 gene; *P* = 0.051). Additional pathways showing nominal significance included the citrate cycle/TCA cycle (hsa00020; 1 gene; *P* = 0.055), signaling pathways regulating pluripotency of stem cells (hsa04550; 2 genes; *P* = 0.057), 2-oxocarboxylic acid metabolism (hsa01210; 1 gene; P = 0.057), SNARE interactions in vesicular transport (hsa04130; 1 gene; *P* = 0.059), biosynthesis of unsaturated fatty acids (hsa01040; 1 gene; *P* = 0.069), and pyruvate metabolism (hsa00620; 1 gene; *P* = 0.084). The top enriched pathways are visualized in Figure 4, and a complete list of tested pathways is provided in Supplementary Table 3.

**FIGURE 5:**
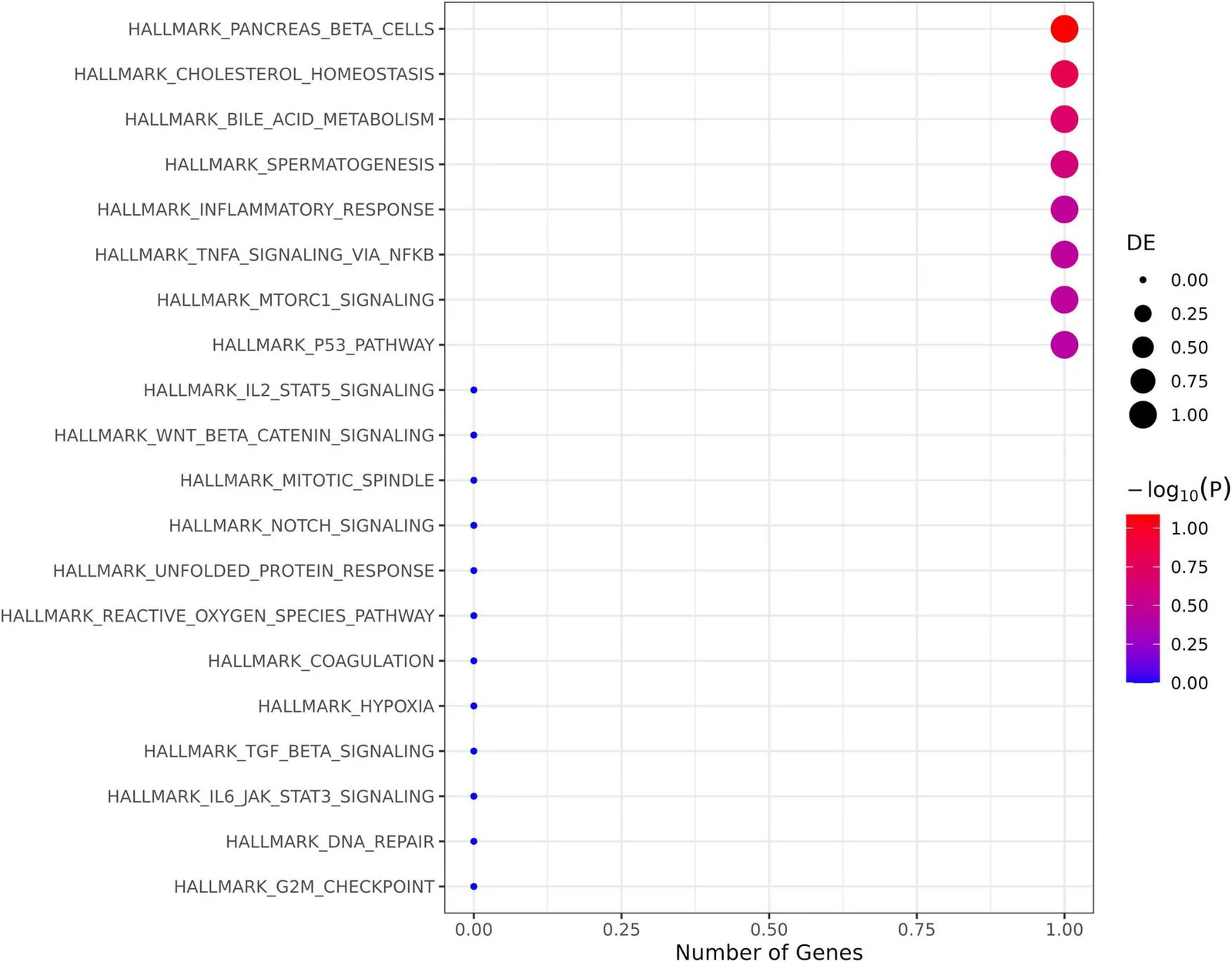
MSigDB Hallmark gene set enrichment for Top 50 DMLs (*P* < 0.0001 threshold) in the intervention group. Dot plot showing the top MSigDB Hallmark gene sets enriched among the top 50 significantly differentially methylated CpG sites. The *x*-axis indicates the number of differentially methylated genes in each gene set, the *y*-axis shows gene set names, dot size represents gene count, and dot color represents the negative log_10_-transformed *P*-value (blue = less significant, red = more significant). Open circles indicate gene sets that did not reach FDR < 0.05 after multiple testing correction. Analysis was performed using the missMethyl gsameth function to account for probe number bias.

Hallmark gene set enrichment analysis identified one gene set showing nominal significance at *P* < 0.1: HALLMARK_PANCREAS_BETA_CELLS (1 differentially methylated gene; *P* = 0.082). Additional gene sets included HALLMARK_CHOLESTEROL_HOMEOSTASIS (1 gene; *P* = 0.152), HALLMARK_BILE_ACID_METABOLISM (1 gene; *P* = 0.202), and HALLMARK_SPERMATOGENESIS (1 gene; *P* = 0.242). Gene sets related to inflammation showed moderate enrichment, including HALLMARK_ INFLAMMATORY_RESPONSE (1 gene; *P* = 0.334) and HALLMARK_TNFA_SIGNALING_VIA_NFKB (1 gene; *P* = 0.345). Enriched Hallmark gene sets for the top 50 DMLs (*P* < 0.0001) are visualized in Figure 5, and complete results are provided in Supplementary Table 4.

#### 676 Top-ranked DMLs (*P* < 0.001, FDR > 0.05) in the intervention group

KEGG pathway enrichment analysis of the 676 DMLs identified at the *P* < 0.001 threshold revealed several nominally enriched pathways (*P* < 0.1, FDR = 1.0 for all KEGG pathways tested). The most significantly enriched pathway was alpha-linolenic acid metabolism (hsa00592; 4 differentially methylated genes; *P* = 0.0031). This was followed by parathyroid hormone synthesis, secretion and action (hsa04928; 9 genes; *P* = 0.030), fatty acid metabolism (hsa01212; 5 genes; *P* = 0.031), vascular smooth muscle contraction (hsa04270; 9 genes; *P* = 0.032), ether lipid metabolism (hsa00565; 4 genes; *P* = 0.038), PI3K-Akt signaling pathway (hsa04151; 18 genes; *P* = 0.041), folate transport and metabolism (hsa04981; 3 genes; *P* = 0.042), PPAR signaling pathway (hsa03320; 5 genes; *P* = 0.049), and biosynthesis of unsaturated fatty acids (hsa01040; 3 genes; *P* = 0.050). Vibrio cholerae infection (hsa05110; 4 genes; *P* = 0.065) and RIG-I-like receptor signaling pathway (hsa04622; 4 genes; *P* = 0.069) were also nominally enriched; infection-related KEGG gene sets of this kind most likely reflect incidental overlap with broadly expressed genes rather than a biologically meaningful finding. Glycolysis/gluconeogenesis (hsa00010; 4 genes; *P* = 0.077) and carbon metabolism (hsa01200; 6 genes; *P* = 0.080) were also nominally enriched.

**FIGURE 6:**
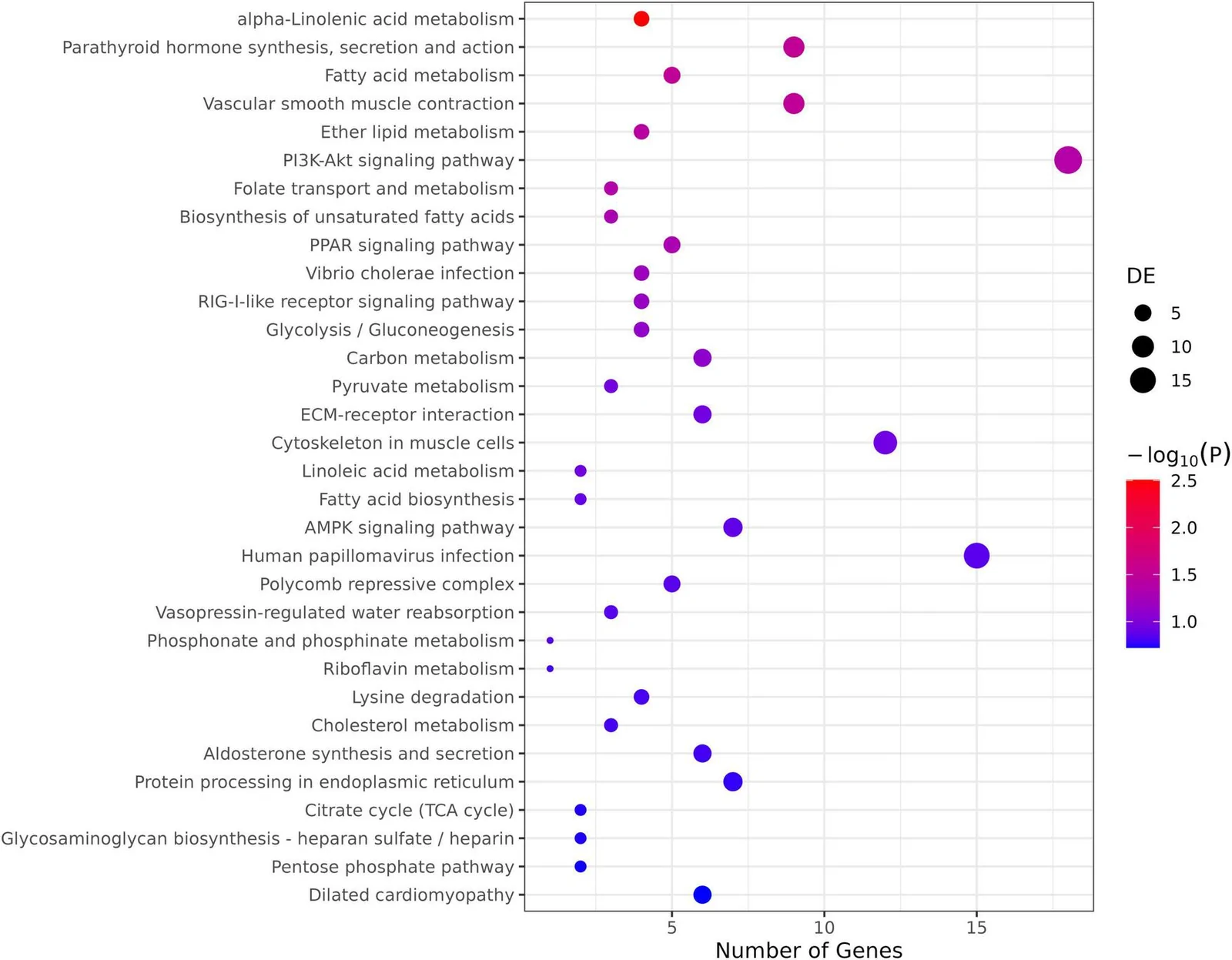
Enriched KEGG pathways for top 676 DMLs (P 0.001 threshold) in the intervention group (P 0.2 Shown). Dot plot showing the top KEGG pathways enriched among 676 significantly differentially methylated CpG sites (P 0.001) in the intervention group. The x-axis indicates the number of differentially methylated genes in each pathway, the y-axis shows pathway names, dot size represents gene count, and dot color represents the negative log_10_-transformed P-value (blue = less significant, red = more significant). Open circles indicate pathways that did not reach FDR 0.05 after multiple testing correction. Analysis was performed using the missMethyl gometh function to account for probe number bias.

Additional enriched pathways (0.1 < *P* < 0.2, FDR > 0.05) included pyruvate metabolism (hsa00620; 3 genes; *P* = 0.112), ECM-receptor interaction (hsa04512; 6 genes; *P* = 0.113), cytoskeleton in muscle cells (hsa04820; 12 genes; *P* = 0.116), linoleic acid metabolism (hsa00591; 2 genes; *P* = 0.117), fatty acid biosynthesis (hsa00061; 2 genes; *P* = 0.129), AMPK signaling pathway (hsa04152; 7 genes; *P* = 0.131), citrate cycle/TCA cycle (hsa00020; 2 genes; *P* = 0.183), glycosaminoglycan biosynthesis - heparan sulfate/heparin (hsa00534; 2 genes; *P* = 0.183), and pentose phosphate pathway (hsa00030; 2 genes; *P* = 0.187). The top enriched pathways are visualized in Figure 6, and a complete list of tested KEGG pathways is provided in Supplementary Table 5.

**FIGURE 7:**
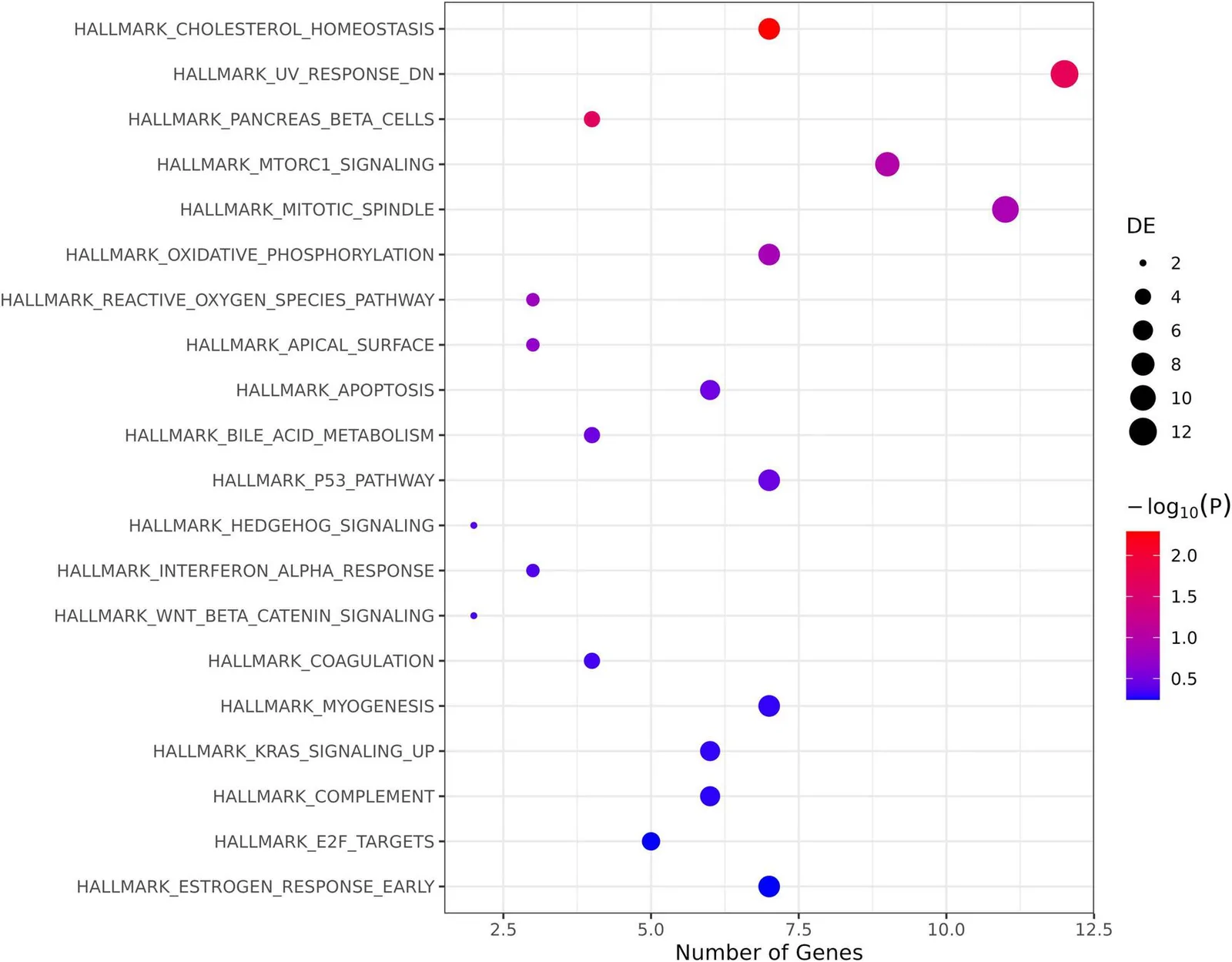
MSigDB Hallmark gene set enrichment for 676 differentially methylated loci (*P* < 0.001 Threshold) in the intervention group. Dot plot showing nominally enriched MSigDB Hallmark gene sets among 676 differentially methylated CpG sites identified in the intervention group (within-individual change, baseline to Week 8; *P* < 0.001, FDR > 0.05). The *x*-axis indicates the number of differentially methylated genes in each gene set; the *y*-axis shows gene set names ordered by significance. Dot size represents the number of differentially methylated genes and dot color represents the negative log_10_-transformed unadjusted *P*-value, with warmer colors (red) indicating greater significance. Open circles indicate gene sets that did not reach FDR < 0.05 after Benjamini-Hochberg correction. Analysis was performed using the missMethyl gsameth function to account for probe-number bias.

Hallmark gene set enrichment analysis identified two gene sets approaching nominal significance. The most enriched gene set was HALLMARK_CHOLESTEROL_HOMEOSTASIS (7 differentially methylated genes; *P* = 0.0051, FDR = 0.26), the lowest FDR observed across all KEGG and Hallmark analyses in this study, though still above the FDR < 0.05 threshold. This was followed by HALLMARK_UV_RESPONSE_DN (12 genes; *P* = 0.018, FDR = 0.37) and HALLMARK_PANCREAS_BETA_CELLS (4 genes; *P* = 0.022, FDR = 0.37). Additional gene sets included HALLMARK_MTORC1_SIGNALING (9 genes; *P* = 0.105), HALLMARK_MITOTIC_SPINDLE (11 genes; *P* = 0.129), HALLMARK_OXIDATIVE_PHOSPHORYLATION (7 genes; *P* = 0.138), and HALLMARK_REACTIVE_OXYGEN_SPECIES_ PATHWAY (3 genes; *P* = 0.171). Enriched Hallmark gene sets for the top 676 DMLs (*P* < 0.001) are visualized in Figure 7, and complete results are provided in Supplementary Table 6.

### Group-by-time interaction analysis

To complement the within-group temporal analyses, we conducted a group-by-time interaction contrast comparing methylation trajectories between treatment and control arms from baseline to week 8. 694 CpG sites met the nominal threshold of *P* < 0.001 and 70 demonstrated significant interaction effects at the *P* < 0.0001 threshold, with 36 positive interactions (treatment group showed greater methylation increases or smaller decreases than controls) and 34 negative interactions. Interaction QQ and volcano plots are presented in Supplementary Figures 2, 3 and complete results are provided in Supplementary Table 7.

#### Comparison of interaction analysis with within-group analyses

Of the 70 interaction CpGs (*P* < 0.0001), 18 (25.7%) overlapped with the 676 CpGs identified in the within-group temporal analysis (2.7% of within-group CpGs), indicating that while the interaction contrast captures a largely distinct set of methylation changes reflecting intervention-specific trajectories, a meaningful minority of interaction CpGs also show detectable within-group temporal change. Two of the 70 interaction CpGs overlapped with the 15 transcription start site-proximal CpGs (Table 1) (2.9%; cg05950123, STX2; cg19367388, UBE2R2; both hypomethylated in the intervention group relative to the control group), and 4 (5.7%) overlapped with the 50 most significant within-group CpGs.

### Functional enrichment of group-by-time interaction CpGs

At nominal *P* < 0.05, Hallmark analysis of all 694 interaction CpGs (*P* < 0.001, FDR > 0.05) identified E2F Targets (11 genes including DIAPH3, MCM4, MYC, RAD50, STMN1; *P* = 0.013) and MYC Targets V2 (4 genes including MYC, PPRC1; *P* = 0.045), implicating cell-cycle transcription factors with known roles in metabolic regulation, mitochondrial biogenesis, and one-carbon folate flux. Extending the discovery threshold to nominal *P* < 0.1 revealed enrichment in Reactive Oxygen Species (ROS) Pathway (4 genes: ABCC1, GCLM, MBP, PDLIM1; *P* = 0.062) and WNT/β-catenin Signaling (4 genes: CTNNB1, MYC, NCOR2, NOTCH4; *P* = 0.080). MYC appeared in both the WNT/β-catenin and E2F Targets gene sets, underscoring its role as a central coordinator of transcriptional and metabolic reprogramming. Among KEGG pathways, glycosaminoglycan biosynthesis – heparan sulfate/heparin (*P* = 0.050) and thyroid hormone signaling pathway (*P* = 0.056) reached nominal significance at *P* < 0.1, suggesting involvement in extracellular matrix remodeling and systemic metabolic regulation.

Focused enrichment analysis of the 70 most significant interaction CpGs (*P* < 0.0001, FDR > 0.05) used the 694 tested interaction CpGs as the background, following methods identical to the within-group analyses. At nominal *P* < 0.05, two KEGG pathways were enriched: Autophagy – animal (hsa04140; SQSTM1/p62, TSC2, VPS39; *P* = 0.014) and ATP-dependent chromatin remodeling (hsa03082; GATAD2A, INO80E, SMARCA4; *P* = 0.050). PI3K/AKT/mTOR Signaling (SQSTM1, TSC2; *P* = 0.046) reached nominal significance in the Hallmark analysis. Extending to *P* < 0.1, mTOR signaling pathway (hsa04150; *P* = 0.073) and ubiquitin-mediated proteolysis (hsa04120; *P* = 0.088) were additionally enriched. Complete enrichment results of the 694 and 70 interaction CpGs are provided in Supplementary Tables 8, 9. Result highlights are visualized in Supplementary Figure 4.

### Differentially methylated region (DMR) analysis

To assess whether co-methylated adjacent CpG sites produce regional signals surviving FDR correction, we applied DMRcate v3.2.1 to the treatment-arm within-group comparison and the group-by-time interaction model. In the within-group analysis (*n* = 18 treatment participants, Baseline vs. Week 8), 128 DMRs were identified at HMFDR < 0.05. Several correspond directly to loci discussed above, including a 9-CpG region spanning the SLC39A7/RXRB locus on chr6 (the top-ranked single-CpG hit), a 5-CpG region at STX2 on chr12, a 6-CpG region at ZNF503 on chr10, and a 4-CpG region at RBMS1 on chr2. Applying the same framework to the group-by-time interaction model (all 38 subjects) yielded 129 DMRs at HMFDR < 0.05, including regions at VPS39 on chr15 (9 CpGs), INO80E/HIRIP3 on chr16 (18 CpGs), and STX2 on chr12 (3 CpGs). The convergence of STX2 in both DMR analyses, and of VPS39 and INO80E/HIRIP3 in the interaction DMR, is consistent with their appearance among the top-ranked single-CpG hits. Because candidate regions were constructed only from CpGs already meeting a nominal single-site threshold, the regional HMFDR values are conditional on this preselection and do not constitute independent genome-wide FDR control. Accordingly, these results should be interpreted as a second-stage assessment of spatial coherence among the top nominal loci rather than as an independent genome-wide DMR discovery analysis. Full DMR results are provided in Supplementary Tables 10, 11.

### Cell composition sensitivity analysis

To assess robustness of the within-group findings to longitudinal shifts in salivary cell composition, we performed a sensitivity analysis excluding the five participants (of 18) with the largest total cell-proportion shift from Baseline to Week 8 (top quartile by summed absolute change across Epi/Fib/IC fractions; *n* = 13 retained). The logFC correlation between the full model (*n* = 18) and the cell-stable subset (*n* = 13) across all 676 top-ranked DMLs in the intervention group (*P* < 0.001) was Pearson r = 0.98 and Spearman r = 0.98, with 100% directional concordance. The reduced hit count in the sensitivity subset at *P* < 0.001 (268 vs. 676) is consistent with expected power loss from the smaller sample rather than a loss of directional signal, confirming that the reported findings are not attributable to cell composition shifts during the intervention.

### Cross-study comparison of dietary intervention methylation signatures

**FIGURE 8:**
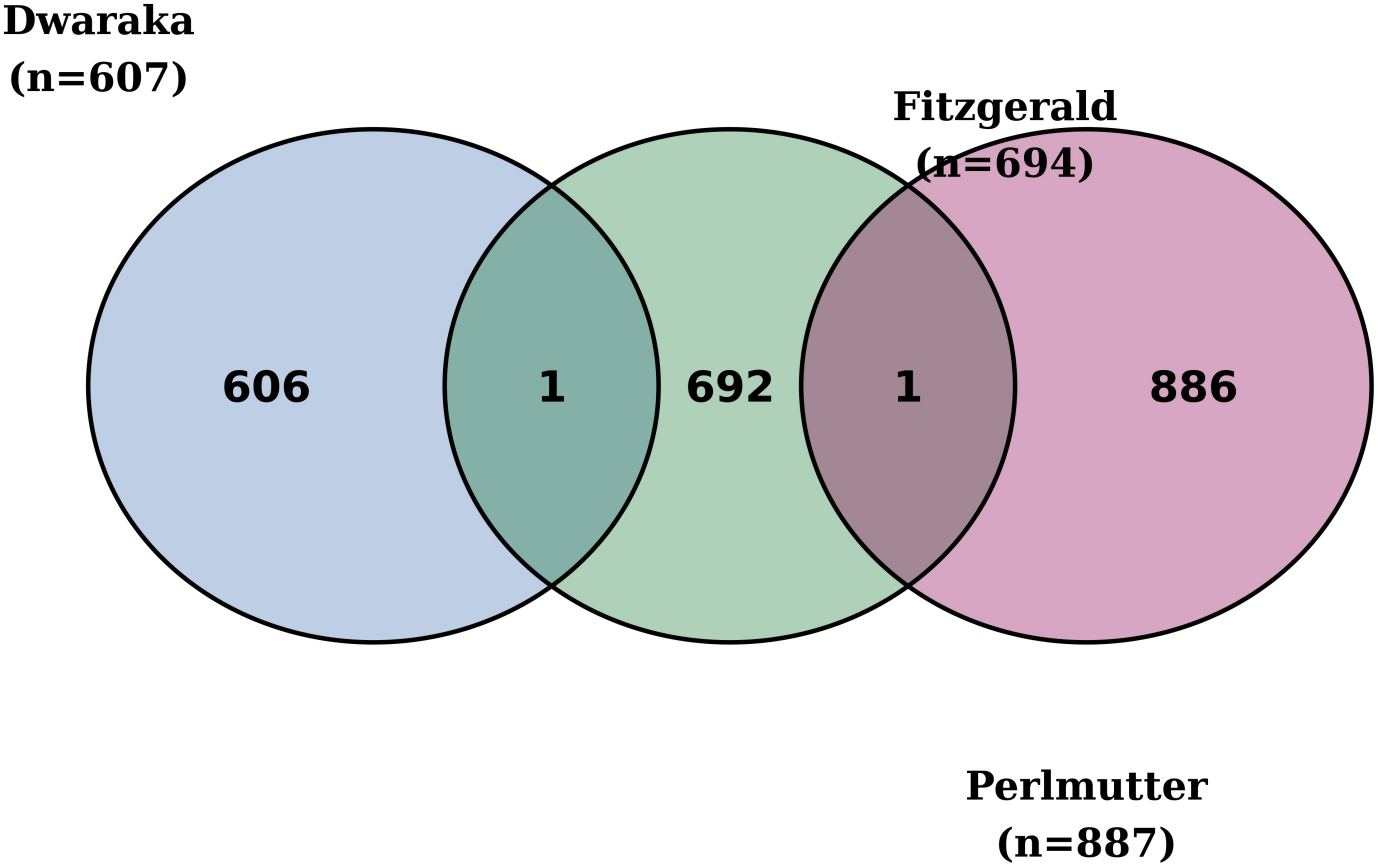
Cross-Study comparison of interaction CpG overlap across three independent dietary intervention EWAS. Venn diagram illustrating the overlap of differentially methylated CpG sites across three independent dietary intervention epigenome-wide association studies. The Fitzgerald set comprises 694 CpGs identified in the group-by-time interaction analysis (*P* < 0.001) from the present study. The Dwaraka set comprises 607 CpGs identified in an 8-week vegan versus omnivore dietary intervention (TwiNS study). The Perlmutter set comprises 887 CpGs identified following 90 days of Tartary buckwheat polyphenol supplementation. Overlap was assessed by exact CpG probe ID matching regardless of direction of methylation change.

To assess cross-study reproducibility of dietary polyphenol methylation signatures, the 694 interaction CpGs (*P* < 0.001) were compared with CpGs from two independent dietary EWAS (Figure 8). Minimal overlap was observed: one CpG was shared between the Fitzgerald and Perlmutter et al. (18) analyses (cg03757262; intergenic; hypomethylated in both; Fitzgerald logFC = −0.020, Perlmutter logFC = −0.200), and one CpG was shared between Fitzgerald and Dwaraka et al. (13) (cg08127021 in LRP5; hypermethylated in Fitzgerald logFC = +0.016 but hypomethylated in Dwaraka logFC = −0.339). No CpGs were common to all three studies, and no CpGs overlapped between the Perlmutter and Dwaraka sets. The discordant direction at the LRP5 locus, a Wnt pathway co-receptor, across studies may reflect context-dependent epigenetic regulation of Wnt signaling by distinct dietary exposures.

## Discussion

This study sought to examine the impact of a DNA methylation-targeted diet and lifestyle intervention on CpG methylation and its potential to influence phenotypic expression using results from a randomized, controlled, clinical pilot trial. The inclusion of a control group in an EWAS analysis is less common and therefore of potential additional interest. The use of nominal significance thresholds of *P* < 0.001 and *P* < 0.0001 was consistent with prior EWAS studies, for hypothesis generation, to examine directionality, and perform enrichment analyses. The absence of FDR-significant loci or pathways should be carefully interpreted, as it could be due to a limitation of statistical power and a reflection of the small sample size and short intervention duration. Additionally, the lack of achieving an FDR < 0.05 pathway enrichment may also suggest that the differentially methylated CpGs are distributed across diverse biological processes rather than concentrated in a few specific pathways.

### Within-group differential methylation analysis

Using the nominal threshold of *P* < 0.001, 676 DMLs were identified in the intervention group versus 286 in the control group. At a stricter threshold of *P* < 0.0001, 50 DMLs were identified in the intervention group versus 13 in the control group. Based on prior analysis of these data that identified the intervention factors most likely to influence epigenetic age (9), we theorize that the deliberate inclusion in the intervention of dietary and lifestyle inputs known to alter epigenetic machinery, and in particular phytonutrients and polyphenols such as those found in green tea, oolong tea, turmeric, garlic, and berries (the components of the intervention found to be most predictive of epigenetic age attenuation in this cohort (9)), contributed to the greater number of DMLs identified in the intervention group compared to controls at both *P*-value thresholds.

#### Exploratory analysis of transcription start site-proximal DMLs

Certain CpG sites may play a greater role in gene expression and phenotype and are therefore of interest for further exploration. Of the 50 DMLs identified at the threshold of *P* < 0.0001, 15 DMLs are in transcription start site-proximal regions of 15 genes. While the clinical implications of these changes are not yet fully understood, differential methylation at promoter- and transcription start site (TSS)-proximal CpGs is considered of particular interest since it is inferred that these sites may have more predictable effects on gene expression and phenotype. For example, in other research using dietary and lifestyle interventions, DNA methylation of the RALBP1 promoter after a 6 month exercise intervention was found to suppress gene transcriptional activity (19). Additionally, maternal folic acid supplementation during pregnancy was found to increase DNA methylation at the promoter region of the imprinted gene regulator ZPF57 in cord blood samples and that increased DNA methylation at this site was associated with reduced ZPF57 transcription (20).

There are further precedents for single-CpG biomarkers with functional relevance. The best characterized is cg05575921 in AHRR, which shows large, dose-dependent hypomethylation in smokers, reverses partially after cessation, and serves as a highly sensitive and specific marker of tobacco exposure across cohorts and ancestries (21, 22). CpGs in the AHRR and F2RL3 loci at cg05575921 and cg03636183 are now widely used as single-site biomarkers of smoking status and cumulative exposure (23). Similarly, the Horvath multi-tissue clock uses 353 individual CpGs, selected by elastic-net regression, to track chronological age across multiple tissues with high accuracy (24). Forensic age predictors frequently rely on one or a small number of CpGs, such as those in ELOVL2 (25). These examples demonstrate that single CpGs, when selected rigorously and validated, can encode stable, biologically meaningful information. Nevertheless, the 15 CpGs explored here along with their TSS-proximal genes (Table 1) will require regional analysis, replication, and functional validation before they can be considered robust intervention-responsive biomarkers.

From an exploratory perspective, the loci identified in this study show notable biological themes. First, there emerge several zinc-related genes. SLC39A7 (ZIP7; cg07019243, hypomethylated) is an endoplasmic reticulum/Golgi zinc transporter. ZIP7-mediated zinc flux has been shown to gate multiple downstream signaling pathways, including growth-factor and metabolic signaling, and its alteration is associated with dysregulated cellular responses to insulin and other cues (26). MTF1 (cg13987712, hypomethylated) encodes metal-responsive transcription factor-1, a zinc-binding factor that senses intracellular Zn^2+^ and binds metal response elements to induce metallothionein and other metal-handling genes. ZNF503 (cg13487762, hypomethylated) encodes a C2H2-type zinc finger transcription factor involved in neural and retinal development (27, 28). cg07019243 is also annotated to RXRB, encoding retinoid X receptor-β, a promiscuous nuclear receptor partner for VDR, RARs, PPARs, and others that collectively regulate lipid and glucose metabolism and inflammatory signaling. The intervention diet emphasized zinc-rich foods and fat-soluble vitamin precursors (5), so reduced methylation at a shared SLC39A7/RXRB promoter-proximal CpG is directionally consistent with enhanced transcriptional responsiveness to zinc and nuclear-receptor ligands, although expression data are not available here.

A second thematic cluster involves epigenetic regulation of development, cell differentiation, pluripotency and aging. JARID2 (cg08895497, hypomethylated) is an accessory component of Polycomb repressive complex 2 (PRC2) that promotes allosteric activation of PRC2 and facilitates H3K27 trimethylation, a canonical repressive mark consistently enriched at age-associated hypermethylated loci (29, 30). JARID2 is a target of core pluripotency since OCT4, SOX2, and NANOG bind its super-enhancer to drive its expression (31), and it physically interacts with SOX2, KLF4, and c-MYC to functionally accelerate reprogramming by suppressing the barrier gene *Arf* and facilitating Nanog promoter demethylation at the early phase of OSKM (OCT4, SOX2, KLF4, c-MYC)-driven reprogramming (32). PAX6 (cg15927696, hypomethylated) is a master developmental transcription factor that continues to influence differentiation and tissue homeostasis in adults, with promoter methylation known to inversely relate to expression in various cellular contexts (33). During embryonic stem cell neural differentiation, PAX6 is upregulated and forms an alternative complex with SOX2 that activates neural gene expression and promotes exit from pluripotency (34).

Several of the remaining genes fall into a broad category of cellular quality control, including proteostasis, DNA damage responses, genome maintenance. These are core “hallmarks of aging” pathways that are highly sensitive to metabolic and redox status (35). BCLAF1 (cg23068057, hypomethylated) encodes BCL2-associated transcription factor-1, which integrates DNA damage signals and senescence. BCLAF1 is up-regulated via the ATM/NEMO/NF-κB pathway under genotoxic stress and is required for full induction of senescence-associated secretory phenotype genes via C/EBPβ (36). UBE2R2 (E2 ubiquitin-conjugating enzyme; cg19367388, hypomethylated), BCAP29 (an endoplasmic reticulum/Golgi trafficking chaperone; cg09189890, hypomethylated), and GEMIN6 (a survival motor neuron [SMN] complex component required for snRNP biogenesis and pre-mRNA splicing; cg07971474, hypomethylated) products participate in the ubiquitin–proteasome system, ER protein handling, and spliceosome assembly, respectively, associated with proteostatic and RNA-processing pathways that are known to deteriorate with age (37).

SSBP2 (cg21249921, hypomethylated) is a single-stranded DNA-binding protein that functions as a tumor suppressor; its promoter is frequently hypermethylated and silenced in multiple malignancies, including prostate and hematologic cancers. In prostate cancer, SSBP2 promoter hypermethylation is observed in a majority of primary tumors and correlates with higher pathologic stage, whereas forced expression suppresses tumor cell proliferation and induces cell-cycle arrest (38). STX2 (cg05950123, hypomethylated), syntaxin 2, is a soluble N-ethylmaleimide-sensitive factor attachment protein receptor (SNARE)-family vesicle trafficking protein that has diverse roles including in insulin granule exocytosis and the release of digestive enzyme zymogen granules from pancreatic acinar cells (39, 40).

One more notable feature of the 15-CpG panel is the enrichment of genes annotated to neuronal and synaptic functions, despite the use of saliva as the sampled tissue. KCNS3 (cg23986335, hypomethylated) encodes the Kv9.3 potassium channel α-subunit, selectively expressed in parvalbumin-positive GABAergic interneurons in the prefrontal cortex (41). IGSF11 (cg14302760, hypermethylated) is a brain- and testis-expressed immunoglobulin superfamily adhesion molecule that binds PSD-95 and regulates AMPA-receptor stability at excitatory synapses (42). PCDH19 (cg00584971, hypermethylated) is an X-linked protocadherin essential for early neuronal adhesion and network formation; pathogenic variants cause PCDH19-related epilepsy, a female-limited developmental epileptic encephalopathy (43). Finally, KLC2 (cg13777862, hypermethylated) encodes kinesin light chain-2, a component of kinesin-1 motors that mediate anterograde axonal transport of mitochondria and other cargos (44). Both insufficient and excessive KLC2 activity can impair axonal transport and have been associated with neurodevelopmental and neurodegenerative phenotypes in experimental models.

### Within-group functional enrichment analysis

In the within-group enrichment analysis, several nominally enriched pathways emerge that align with biologically relevant processes.

In the analysis of the 50 top-ranked DMLs (*P* < 0.0001) in the intervention group alone, enrichment of metabolic pathways, particularly those involved in lipid metabolism (lipoic acid metabolism, alpha-linolenic acid metabolism, biosynthesis of unsaturated fatty acids) and energy metabolism (citrate cycle/TCA cycle, 2-oxocarboxylic acid metabolism, pyruvate metabolism), suggests potential involvement of these CpGs in metabolic regulation. The nominal enrichment of the pancreatic beta cell gene set and the maturity onset diabetes of the young pathway is also noteworthy given their relevance to glucose homeostasis and diabetes pathophysiology. Nominal enrichment of signaling pathways regulating pluripotency of stem cells (2 genes, PAX6 and JARID2; *P* = 0.057) is notable given established roles for both genes in this network and their prominence in the top ranked genes located at transcription start site-proximal regions in this study (Table 1).

In the analysis of the 676 top-ranked DMLs (*P* < 0.001) in the intervention group, several themes emerge. The first is lipid/fatty acid metabolism: consistent enrichment of lipid-related pathways (alpha-linolenic acid metabolism, *P* = 0.0031; ether lipid metabolism, *P* = 0.038; fatty acid metabolism, *P* = 0.031; PPAR signaling pathway, *P* = 0.049; biosynthesis of unsaturated fatty acids, *P* = 0.050, linoleic acid metabolism, *P* = 0.117) suggests involvement in membrane composition, lipid signaling, and metabolic regulation relevant to cellular energy metabolism and inflammatory signaling via DNA methylation. The second is cholesterol homeostasis and stress response: HALLMARK_CHOLESTEROL_HOMEOSTASIS (7 genes, *P* = 0.0051, FDR = 0.26) was the most enriched Hallmark gene set identified in this analysis, the closest any pathway came to surviving FDR correction in this study, followed by HALLMARK_UV_RESPONSE_DN (12 genes, *P* = 0.018) and HALLMARK_PANCREAS_BETA_CELLS (4 genes, *P* = 0.022) - the latter also nominally enriched in the more stringent 50-CpG analysis, a consistency signal across significance thresholds. The third is central metabolism: nominal enrichment of glycolysis/gluconeogenesis, the citrate (TCA) cycle, the pentose phosphate pathway, and carbon metabolism suggests coordinated regulation of core metabolic pathways through DNA methylation. The fourth is signal transduction: enrichment of the PI3K-Akt signaling pathway (18 genes, *P* = 0.041), the largest gene set among the nominally enriched pathways, together with parathyroid hormone synthesis, secretion and action and vascular smooth muscle contraction pathways, may reflect broader hormone- and growth-factor-responsive methylation changes. KEGG PI3K-Akt signaling pathway includes the mTOR signaling pathway, a critical regulator of cellular metabolism and aging (45). Lastly, nominal enrichment of folate transport and metabolism is consistent with the high content of dietary folates in the intervention diet.

Compared to the more stringent analysis of 50 highly significant CpGs (*P* < 0.0001), the expanded analysis of 676 CpGs (*P* < 0.001) demonstrated: (1) Substantially more differentially methylated genes within enriched pathways (e.g., 18 genes in the PI3K-Akt signaling pathway vs. a maximum of 2 genes per pathway in the 50-CpG analysis); (2) A consistent theme of lipid and fatty acid metabolism enrichment across both thresholds - alpha-linolenic acid metabolism strengthened from *P* = 0.049 (1 gene) at the 50-CpG threshold to *P* = 0.0031 (4 genes) at the 676-CpG threshold; (3) A cross-threshold consistency signal in the Hallmark analysis: HALLMARK_PANCREAS_BETA_CELLS was the top nominal hit at 50 CpGs (*P* = 0.082) and remained among the top hits at 676 CpGs (*P* = 0.022), and HALLMARK_CHOLESTEROL_HOMEOSTASIS was nominally enriched at both thresholds (*P* = 0.152 at 50 CpGs; *P* = 0.0051 at 676 CpGs); (4) Persistent lack of an FDR < 0.05 finding, consistent with differentially methylated CpGs being distributed across diverse biological processes rather than concentrated in a few specific pathways.

### Group-by-time interaction analysis

694 CpGs were identified as meeting the nominal *P* < 0.001 threshold in the interaction analysis and a further 70 met the *P* < 0.0001 threshold. The 25.7% overlap between interaction CpGs and within-group temporal CpGs suggests that, while each contrast captures largely distinct methylation changes, there are a meaningful minority of CpGs that are represented in both analyses.

Nominally enriched pathways in the interaction analysis revealed biologically coherent themes centered on nutrient sensing, transcriptional regulation, and cellular homeostasis. Among the broader 694-CpG set, E2F Targets and MYC Targets V2 (both *P* < 0.05) implicate transcription factors whose metabolic functions extend beyond proliferation, including regulation of glucose metabolism, mitochondrial biogenesis, and one-carbon folate flux. These pathways are directly relevant to a methylation-supportive dietary intervention. The ROS Pathway and WNT/β-catenin Signaling, at nominal *P* < 0.1, share mechanistic connectivity via MYC, a downstream effector of WNT signaling that also appeared among E2F Targets. These biological themes are retained across both *P* < 0.05 and *P* < 0.1 nominal thresholds. This within-analysis thematic stability provides a form of internal replication that strengthens interpretation despite the absence of FDR-adjusted significance.

For the focused 70-CpG set, autophagy and mTOR pathways showed the most robust nominally enriched signal, with TSC2 and SQSTM1/p62 appearing consistently at both *P* < 0.05 and *P* < 0.1, further supporting their candidacy as core intervention-responsive epigenetic loci. The convergence of TSC2 and SQSTM1/p62 across autophagy, PI3K/AKT/mTOR, and mTOR pathways at both *P* < 0.05 and *P* < 0.1 thresholds provides convergent evidence for their central role in intervention-responsive epigenetic change. TSC2 functions as a critical negative regulator of mTORC1, integrating nutrient and stress signals to balance anabolic growth with autophagic catabolism; SQSTM1/p62 serves as a selective autophagy receptor and signaling scaffold linking autophagy, oxidative stress, and nutrient sensing. These pathways represent evolutionarily conserved nutrient-sensing mechanisms modulated by caloric restriction, polyphenol exposure, and other longevity-associated dietary interventions, providing a plausible mechanistic substrate for the epigenetic age attenuation previously reported in this cohort (5, 10, 46, 47). Future work integrating DNA methylation with transcriptomic, proteomic, and metabolomic profiling will be essential to establish whether these nominally enriched epigenetic signatures are functionally coupled to gene expression and measurable metabolic outcomes.

### Differentially methylated region (DMR) analysis

Regional analysis using DMRcate identified 128 within-group DMRs and 129 interaction-specific DMRs at HMFDR < 0.05. FDR correction should be interpreted carefully: because candidate regions were built exclusively from CpGs already meeting a nominal single-site threshold (*P* < 0.001), the regional FDR control operates over a substantially smaller and pre-filtered test space than the genome-wide single-CpG analyses. In the within-group analysis, several regions map directly onto loci already highlighted in the single-CpG results. Most prominently, the 9-CpG region spanning SLC39A7/RXRB on chr6 (zinc-transport/nuclear-receptor signal) incorporates the single most significant CpG in the dataset (cg07019243). The DMR at ZNF503 (chr10, a zinc finger transcription factor) similarly reinforces a TSS-proximal gene reported in Table 1. In the interaction analysis, VPS39 and INO80E/HIRIP3 were identified as DMRs (9 and 18 CpGs, respectively). Both genes were also represented among the top-ranked 70 interaction CpGs and among the genes driving nominally enriched KEGG pathways for autophagy (VPS39, alongside SQSTM1/p62 and TSC2) and ATP-dependent chromatin remodeling (INO80E, alongside GATAD2A and SMARCA4) in the focused enrichment analysis. Together, SLC39A7, ZNF503, and MTF1 (with the same underlying zinc-sensing biology, although MTF1 did not itself yield a DMR) form a thematically consistent cluster.

### Biological plausibility and hallmarks of aging

Mechanistically, these DNA methylation shifts are plausibly linked to key features of the intervention. The diet was explicitly formulated to support one-carbon metabolism and S-adenosylmethionine (SAM) synthesis via folate, choline/betaine, and B-vitamin intake, providing the primary methyl donor for DNMT activity. Simultaneously, the diet was intentionally rich in phytochemicals, and especially polyphenols, known to modulate DNMTs and histone deacetylases (HDACs) and a group of polyphenol-dense foods (green tea, oolong tea, rosemary, garlic, and berries) were previously identified as the portion of the intervention most predictive of epigenetic age attenuation after adjusting for weight change and baseline epigenetic age acceleration (5, 9). Specifically, epigallocatechin-3-gallate (EGCG) from green tea directly inhibits DNMT1 and reactivates methylation-silenced tumor-suppressor genes in cancer cell lines (48). Curcumin interferes with DNMT1 activity and acts as an HDAC modulator, leading to locus-specific hypomethylation and increased histone acetylation (49, 50). Sulforaphane from cruciferous vegetables functions as an HDAC inhibitor, altering both histone acetylation and DNA methylation at target promoters (51, 52). Together with improved zinc intake and zinc-dependent MTF1 signaling, these bioactives may create an epigenetic milieu in which both hyper- and hypomethylated promoters become more plastic and responsive to physiological signals. The predominance of hypomethylation (12 of 15 CpGs), particularly at genes involved in tumor suppression (SSBP2), development and pluripotency including via PRC2 (JARID2, PAX6), and proteostasis/cellular quality control (GEMIN6, BCAP29, UBE2R2), is consistent with the notion that age-related promoter hypermethylation at these loci might be at least partly reversible through targeted nutritional and lifestyle interventions.

Epigenetic alteration is one of the hallmarks of aging (35), and many of the methylation changes identified in this EWAS occur at CpG sites annotated to genes or pathways implicated in other hallmarks of aging: nutrient sensing via MTF1 and SLC39A7, genomic stability via BCLAF1 and SSBP2, proteostasis via UBE2R2 and GEMIN6, and cellular communication via synaptic genes like KCNS3. Functional enrichment analysis identified potential impacts on mitochondrial function, inflammatory responses, hormonal signaling, and structural tissue integrity such as tight junction pathways. These findings are extended by the group-by-time interaction analysis: enrichment of pathways related to autophagy, mTOR, and WNT/β-catenin signaling represent evolutionarily conserved mechanisms that regulate cellular metabolism, growth, renewal, and survival. Together, these findings add to previous analyses of the mechanistic effects of polyphenols on all the hallmarks of aging (10).

### Cross-study comparison

Complementary evidence for the impact of polyphenol-rich interventions on the human epigenome is provided by Perlmutter et al. (18) who reported that a high polyphenol supplement containing Tartary buckwheat (*fagopyrum tataricum*) taken for 90 days resulted in 887 DMLs (*n* = 40, *P* < 0.001) (18). In addition, an EWAS analysis by Dwaraka and colleagues of data from the Twins Nutrition Study (TwiNS) found that an 8-week plant-based (vegan) diet resulted in 607 DMLs compared to 494 DMLs for the omnivore diet cohort (*P* < 0.001) (13). Despite shared dietary features, CpG-level overlap with the present study’s findings was minimal. This minimal concordance may reflect fundamental differences in statistical model (interaction contrast vs. main effect EWAS), intervention type (Mediterranean polyphenol-targeted diet vs. buckwheat supplement vs. whole-food vegan diet), study population, and duration, consistent with the high study-specificity of diet-related methylation signatures (1). It is also consistent with the broader observation that diet-related methylation signatures show limited CpG-level replication across independent cohorts (1), potentially reflecting genuine intervention specificity and sensitivity to population, dietary context, analytical model, and duration. The discordant direction of the shared LRP5 CpG (a Wnt pathway co-receptor) across interventions, for example, illustrates this context-dependence.

Longer intervention durations in larger cohorts may yield more substantive epigenomic impacts that better survive multiple testing corrections. For example, in the DIRECT PLUS trial (*n* = 256, 18 months), the green-MED diet, providing an additional 1240 mg polyphenols daily through walnuts, green tea, and Mankai duckweed, resulted in 1573 FDR-significant differentially methylated regions (DMRs) (53). This was substantially more than the 177 for the MED diet group (with added walnuts) and 377 in the healthy dietary guidelines group. That the polyphenol-enriched arm substantially outperformed the standard Mediterranean diet group suggests that polyphenol load may influence the magnitude of epigenetic response in a dose- or threshold-dependent manner. A comparison of studies using polyphenol-rich interventions is provided in Table 2.

**TABLE 2:** Comparison of EWAS studies using polyphenol-rich interventions. Note that these studies were conducted with different effect sizes (e.g., varying sample sizes, durations, samples, and statistical thresholds), so direct numerical comparisons of results are not standardized.

Study	Intervention	*N* Duration	Sample	N DML/DMR (threshold)	Key findings
Current study	Methylation-targeted diet and lifestyle	*N* = 38 8 weeks	Saliva	676 DML (*P* < 0.001) 50 DML (*P* < 0.0001)	Epigenetic age attenuation Metal/nutrient sensing Chromatin regulation Proteostasis Genome stability Synaptic/neuronal function Lipid metabolism Hormonal signaling
TwiNS (13)	Vegan Omnivore	*N* = 42 8 weeks	Blood	607 DML (*P* < 0.001) – vegan 494 DML (*P* < 0.001) – omnivore	Vegan: Epigenetic age attenuation Telomere increase Epigenetic biomarker proxies
High-Polyphenol Supplement (18)	Tartary buckwheat	*N* = 40 90 days	Blood	887 DML (*P* < 0.001)	Immune-related Ceramide kinase activity COP9 activity Photoreceptor cell differentiation
DIRECT PLUS (53, 55)	Green-MED MED HDG	*N* = 256 18 months	Blood	1573 DMR (FDR < 0.05) - Green-MED 377 DMR (FDR < 0.05) - HDG 177 DMR (FDR < 0.05) - MED	Green-MED: Lipid metabolism Protein kinase signaling Circadian entrainment Calcium signaling Wnt signaling cAMP signaling Adipocytokine signaling

## Limitations

It is important to emphasize the limitations of this work and interpret these findings cautiously. Because this secondary analysis included only 38 participants and an 8-week intervention, it was statistically underpowered to detect small methylation changes at ∼866,000 individual CpGs after correction for the number of tests on the EPIC array and the additional burden of pathway-level testing. Under these conditions, failure to reach FDR < 0.05 for single CpGs or enriched pathways is not surprising, and a non-trivial fraction of the nominally significant findings reported here are likely to represent chance associations. Even though the DMR analysis identified several regions at HMFDR < 0.05, DMR findings in small cohorts should be interpreted cautiously since adjacent CpGs tend to co-vary and regional signals may be amplified by the smoothing kernel, and because regional FDR control operates over a pre-filtered subset of sites. For these reasons, our findings should be regarded as biologically plausible, hypothesis-generating candidates rather than definitive intervention targets.

The data derive from bulk saliva, which reflects a mixture of buccal epithelial cells and leukocytes; shifts in cell-type composition over the 8-week intervention could contribute to apparent methylation changes. This concern is partially addressed by the statistical design of this study, which included cell composition as a covariate, and by a sensitivity analysis excluding the five participants with the largest cell-composition shift (*n* = 13 retained): logFC estimates for top-ranked CpGs were highly concordant with the full cohort (Pearson *r* = 0.98, 100% directional concordance), suggesting the within-group findings are not primarily attributable to cell-type drift. This analysis, however, tested consistency of effect estimates among already-selected CpGs rather than independently confirming CpG selection, and was not extended to the interaction model; robustness of the interaction-specific findings to cell composition therefore remains untested. Single-cell methylome profiling could provide more resolution to the cell specific signal in future work.

Tissue specificity is also a major caveat: while certain epigenetic clock CpGs show cross-tissue concordance, most promoter methylation changes are strongly cell-type dependent, and it is not known to what extent saliva methylation at the genes discussed here parallels methylation in liver, muscle, adipose, or brain. We also did not measure gene expression, protein abundance, histone modifications, or functional endpoints (e.g., cognitive testing, detailed metabolic phenotyping) in this sub-analysis; any inference about transcriptional up- or down-regulation based on promoter methylation direction is therefore probabilistic and must be interpreted as such.

Finally, the intervention was multimodal, combining diet, exercise, sleep, and stress-management; the present data cannot attribute the observed methylation changes to any single component of the intervention, including polyphenol intake specifically, nor determine individual component vs. combined effects. This is illustrated by the recent DO-HEALTH randomized controlled trial (*n* = 777), a 3-year study of vitamin D, omega-3, and exercise, in which omega-3 alone slowed several DNA methylation clocks (PhenoAge, GrimAge2, and DunedinPACE), with only modest additional benefit from combining all three factors (54). Factorial designs with substantially larger samples would be required to partition individual contributions and explore whether complex dietary and lifestyle interventions engage distinct methylation mechanisms compared with single-nutrient studies. Validation in larger cohorts and functional studies would be needed to establish causality and biological relevance.

## Conclusion

In this exploratory secondary EWAS of paired salivary EPIC methylation profiles (*n* = 38; 18 treatment, 20 control), an 8 week, polyphenol-dense, DNA methylation–supportive diet and lifestyle intervention was associated with a greater number of nominal within-individual methylation changes than observed in the control arm. Among the highest-ranked loci, transcription start site–proximal CpGs mapped to genes with plausible relevance to aging biology, including nutrient/metal sensing and stress-responsive regulation, development and pluripotency, and pathways related to proteostasis and genome maintenance. A complementary group-by-time interaction analysis identified 70 intervention-specific DMLs. Enrichment analyses also suggested (nominal) clustering within lipid, core energy metabolism, and nutrient sensing pathways. A complementary DMR analysis identified 128 within-group and 129 interaction-specific regions at HMFDR < 0.05, providing regional co-methylation evidence consistent with several of the loci highlighted in the single CpG analyses. Cross-study comparison with other EWAS in polyphenol-rich interventions revealed minimal CpG-level overlap, indicating that dietary methylation signatures are intervention- and context-specific, a finding with important implications for the reproducibility and generalizability of dietary EWAS. Importantly, none of the locus-level or pathway-level findings survived FDR correction. These findings should therefore be interpreted as hypothesis-generating and warrant replication in larger, well-powered cohorts, as well as multi-omic follow-up including RNA-seq, proteomics, and functional readouts.

## Data Availability

The original contributions presented in this study are included in this article/Supplementary material, further inquiries can be directed to the corresponding author.
